# Temporal Change in Renoprotective Effect of Tolvaptan on Patients with Heart Failure: AURORA Study

**DOI:** 10.3390/jcm11040977

**Published:** 2022-02-13

**Authors:** Masami Nishino, Yasuyuki Egami, Akihiro Tanaka, Shodai Kawanami, Hiroki Sugae, Kohei Ukita, Akito Kawamura, Hitoshi Nakamura, Yutaka Matsuhiro, Koji Yasumoto, Masaki Tsuda, Naotaka Okamoto, Yasuharu Matsunaga-Lee, Masamichi Yano, Jun Tanouchi

**Affiliations:** Division of Cardiology, Osaka Rosai Hospital, Sakai 591-8025, Japan; egmyasuyuki@gmail.com (Y.E.); t_anarchy714@yahoo.co.jp (A.T.); sk08014361174@icloud.com (S.K.); hirokisugae.221@gmail.com (H.S.); u9017k@gmail.com (K.U.); aktkwmr1990@gmail.com (A.K.); simple_h_4_19@yahoo.co.jp (H.N.); yutaka1988handai@yahoo.co.jp (Y.M.); yasu87423@gmail.com (K.Y.); masaki.1901@gmail.com (M.T.); okamo10nao@yahoo.co.jp (N.O.); sumomo0304@gmail.com (Y.M.-L.); myano0820@gmail.com (M.Y.); jtanouch@gmail.com (J.T.)

**Keywords:** heart failure, diuretics, tolvaptan, renal function

## Abstract

(1) Background: It has been reported that tolvaptan (TLV) has a renoprotective effect in acute decompensated heart failure (ADHF) patients, but whether this effect is continued for a long time is unclear. Thus, we evaluated the time course of the renoprotective effect of TLV, in addition to the prognosis, in ADHF patients. (2) Methods: We investigated 911 ADHF patients from the AURORA (Acute Heart Failure Registry in Osaka Rosai Hospital) registry. After propensity score matching, 58 patients who started to receive TLV at least two days after the hospitalization (TLV group) and 58 who did not (non-TLV group) were examined. We compared the changes in the creatinine (Cr) and estimated glomerular filtration rate (eGFR) between baseline and each time point (five days, discharge, and one year) as the index of the renoprotective effect, and rate of rehospitalizations and all-cause mortality for one year between the two groups. (3) Results: The change in Cr and eGFR levels was significantly higher in the TLV group than the non-TLV group five days after admission but the difference between the two groups gradually diminished. A Kaplan–Meier analysis showed that the survival and rehospitalization rates in the TLV and non-TLV groups were similar up to one year. (4) TLV revealed a temporal change in the renoprotective effect, which may be correlated with no long-term beneficial effect of TLV.

## 1. Introduction

Acute decompensated heart failure (ADHF) is one of the leading causes of hospital admissions, and the standard treatment is usually pharmacologic involving loop diuretics [1]. However, loop diuretics may lead to a worsening renal function (WRF), which in turn can lead to increased morbidity and mortality [2]. There has been growing interest in alternative strategies to manage the volume overload in ADHF patients.

Tolvaptan (TLV), an oral selective vasopressin 2 receptor antagonist, was approved in Japan in December 2010 for the treatment of excess body fluid not responding to loop diuretics and has been established in Japan for the treatment of a volume overload for heart failure (HF) in contrast to the negative evaluation in the USA and EU countries. Especially, a recent Japan large-scale survey, which consisted of 257,812 patients hospitalized because of HF between April 2008 and November 2018 in Japan, showed that TLV was prescribed within two days of hospitalization in >50% of HF cases since 2015 [3]. In addition, it has been reported that TLV can achieve decongestion with a lower risk of WRF [4]. Regarding the short-term or in-hospital effects of TLV, recent randomized control studies have shown that TLV increased the urine volume and ameliorated the dyspnea by one day post-admission, followed by an increase in the serum sodium concentration and a reduction in body weight [5]. On the contrary, the long-term effects of TLV for HF remain controversial. The first prospective and randomized long-term trial, EVEREST, resulted in neither an improvement nor reduction in the survival nor in the combined endpoint of cardiovascular mortality or HF hospitalizations for a 2-year TLV therapy [6]. However, recent long-term studies using TLV, particularly in Japan, revealed partially favorable TLV effects on HF patients [7,8]. Additionally, whether the renoprotective effect of TLV can be continued or not for a long-term period is unclear.

Few studies have reported the short and long-term TLV effects on HF patients consecutively. Thus, in this study, we evaluated the temporal change in the renoprotective effects of TLV, in addition to the prognosis, in HF patients by propensity score matching to adjust for the patient background continuously in the same patients.

## 2. Methods

### 2.1. Study Population

**Figure 1:**
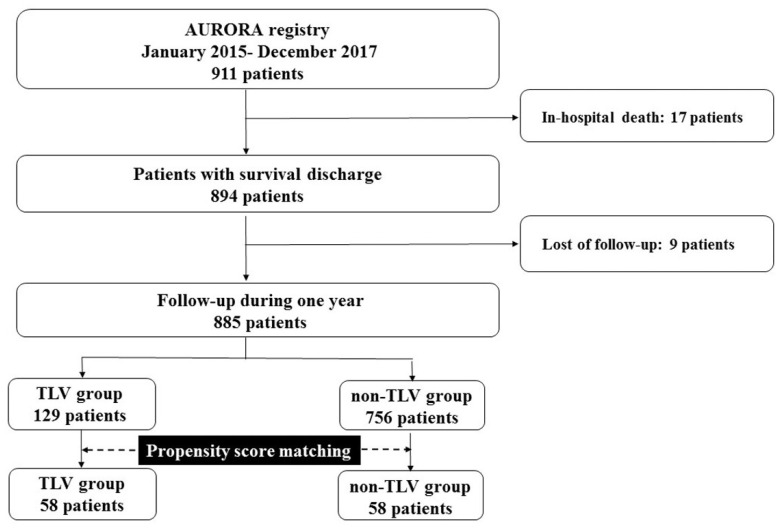
Patient study flow chart. AURORA, Acute Heart Failure Registry in Osaka Rosai Hospital; RH, rehospitalization; TLV, tolvaptan.

AURORA (Acute Heart Failure Registry in Osaka Rosai Hospital) is a single-center registry that collects consecutive ADHF patients who need hospitalizations for treatment at Osaka Rosai Hospital (UMIN-CTR ID: UMIN000045096). We investigated consecutive HF patients who were admitted for ADHF between January 2015 and December 2017 from the AURORA study. According to the Framingham criteria [9], ADHF was diagnosed with at least two major criteria in conjunction with two minor criteria being met. Among them, we excluded the patients who died during hospitalization and who were lost to follow-up one year after discharge (Figure 1). We divided these patients into two groups, the TLV group who started to receive TLV at least 2 days after the hospitalization and non-TLV group who did not receive TLV. In our hospital, early initiation of TLV (within 2 days following the admission) was performed because it has been reported that an early TLV administration is correlated with a better TLV response and better prognosis [5,10]. We performed propensity score matching to adjust for the patient background and HF severity of the patients in the TLV group with those in the non-TLV group on the basis of the age, serum sodium, creatinine (Cr), and plasma B-type natriuretic peptide (BNP) levels, loop diuretic dose, systolic blood pressure, and heart rate [11]. All patients gave a detailed informed consent, and the study protocol was approved by the hospital’s institutional review board. The procedure was in accordance with the ‘Declaration of Helsinki’ and the ethical standards of the responsible committee on human experimentation. This study was granted an exemption from requiring ethics approval by the Osaka Rosai Hospital Ethics Committee because this study was a retrospective observational study, and the permission for using the clinical data was obtained from all study patients on admission.

### 2.2. Data Collection

We collected the data on the following demographic and clinical variables during the hospitalization. We evaluated the age, gender, hypertension, dyslipidemia, diabetes mellitus, chronic kidney disease, history of smoking, past history of an HF admission, length of stay, systolic/diastolic blood pressure and body weight, electrocardiogram (ECG) markers including the heart rate, atrial fibrillation, and QRS duration, laboratory data including the C-reactive protein, BNP, Cr, estimated glomerular filtration rate (eGFR), albumin, sodium, potassium, and uric acid levels, echocardiographic parameters including the left ventricular end-diastolic and systolic dimension (LVDd and LVDs), left ventricular ejection fraction (LVEF), left atrial dimension (LAD), mitral valve regurgitation (MR), aortic valve regurgitation, and tricuspid valve regurgitation, and medications including β blockers, angiotensin-converting enzyme inhibitors (ACEI), angiotensin II receptor blockers (ARB), mineral corticoid receptor antagonists (MRA), loop diuretics, and TLV. The laboratory data, including the Cr and eGFR at 1-, 3-, and 5-days post-admission, just before discharge, and one year after discharge, were evaluated. We also evaluated the dose of the loop diuretics and TLV. The loop diuretic dose was calculated using a furosemide equivalent, which was defined as furosemide 20 mg being equivalent to torasemide 4 mg or azosemide 30 mg. Hypertension was defined as a history of a diagnosis of or treatment for hypertension. Diabetes mellitus was defined as meeting the World Health Organization criteria for diabetes or receiving treatment for diabetes. MR, aortic regurgitation, and tricuspid regurgitation were defined as moderate or severe regurgitation.

We compared the above-mentioned parameters between the TLV group and non-TLV group. As previously mentioned, we performed propensity score matching to adjust for the patient background and HF severity of the patients between the TLV-group and non-TLV group.

To evaluate the short and long-term renoprotective effects of TLV, we calculated the changes in the Cr and eGFR levels between baseline and each time point (5 days, discharge, and one year) as the index of the renoprotective effect. We also compared the incidence of WRF, which was defined as an increase in the serum Cr level of >0.3 mg/dL by 5 days post-admission in this study [12], between the TLV and non-TLV group. In addition, to analyze the clinical outcomes of the TLV treatment we compared the rate of rehospitalizations due to worsening HF and all-cause mortality between the two groups.

### 2.3. Statistical Analysis

JMP 15 statistical software (SAS Institute Inc., Cary, **NC**, USA) was used for the statistical analyses. Continuous variables are reported as the mean ± standard deviation or as the median (interquartile range) for those that were not normally distributed. Continuous variables were compared using a student’s *t*-test if normally distributed or the Wilcoxon rank-sum test if the distribution assumption was not met. Categorical data were expressed as the number (percentage) and were compared using Fisher’s exact test. The differences for the one-year all-cause mortality and HF rehospitalizations between the TLV and non-TLV groups were estimated using Kaplan–Meier curves. A value of *p* < 0.05 was considered to be statistically significant.

## 3. Results

### 3.1. Study Cohort

During the study period, there were 911 ADHF patients who were admitted to have their HF managed in our hospital from the AURORA registry. Among them, we excluded 17 patients because they died during hospitalization. In addition, we also excluded nine patients due to being lost to follow-up after discharge. Accordingly, we investigated the residual 885 patients (Figure 1).

In those 885 study patients, the TLV group consisted of 129 patients and the non-TLV group of 756 patients. After a propensity matching, the analysis was restricted to 116 patients, 58 in the TLV group (50%) and 58 in the non-TLV group (50%).

### 3.2. Patient Characteristics

In the overall cohort, the following patient characteristics were differently distributed in the TLV group and non-TLV group. The TLV group was more likely to have hypertension, CKD, a past history of an HF admission, longer length of stay, lower systolic/diastolic blood pressure, lower heart rate, higher BNP, lower hemoglobin, higher Cr, lower eGFR, lower sodium, higher potassium, larger LVDd/LVDs, lower LVEF, lower grade of MR, higher incidence of β blocker, MRA and loop diuretics, lower incidence of ACEI/ARB, and higher dose of loop diuretics (Table 1). After the propensity-matching, the baseline characteristics were equally distributed except for hypertension, a past history of an HF admission, diastolic blood pressure, and ACEI/ARB use despite having been matched for the propensity scores (Table 1). In the matched cohort, the mean dose of TLV in the TLV group was 11.5 ± 4.9 mg/day (median dose, 15 mg/day). The patients in the TLV group started to receive TLV at an initial dose of 3.75–7.5 mg/day within two days post-admission, and it was increased until the final dose of the TLV, which was decided according to the symptoms and biomarkers by each attending physician before discharge. The patients continued to receive the same dose of TLV for one year after discharge. As a result, 31 patients in the TLV group (53.4%) received 15 mg/day of TLV and 23 in the TLV group (39.7%) received 7.5 mg/day of TLV (3 patients received 3.75 mg/day and one patient received 30 mg/day).

Continuous data are presented as median (interquartile range). Categorical variables are presented as numbers (percentage). ACEI, angiotensin-converting enzyme inhibitor; AF, atrial fibrillation; AR, atrial valve regurgitation; ARB, angiotensin II receptor blocker; BNP, brain natriuretic peptide; CRP, C reactive protein; eGFR, estimated glomerular filtration rate; HF, heart failure; LAD, left atrial diameter; LVDd, left ventricular end-diastolic diameter; LVDs left ventricular end-systolic diameter; LVEF, left ventricular ejection fraction; MR, mitral valve regurgitation; MRA mineralocorticoid antagonist; TLV, tolvaptan; TR, tricuspid valve regurgitation.

### 3.3. Temporal Change in Renoprotection between TLV and Non-TLV Groups

**Figure 2:**
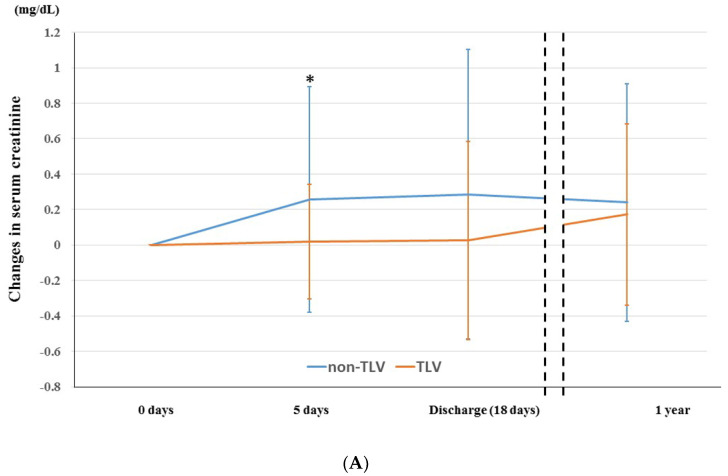

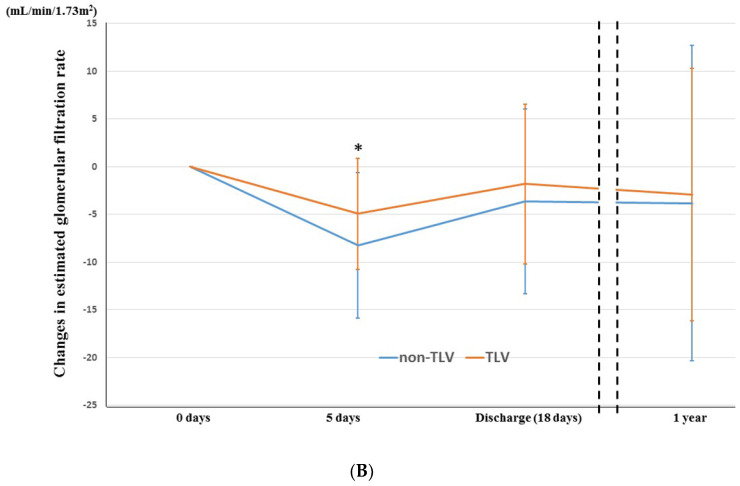
(**A**): Changes in the serum creatinine (Cr) between baseline and each time point (five days post-admission, discharge, and one-year) in the TLV and non-TLV groups. (**B**): Changes in the estimated glomerular filtration rate (eGFR) between baseline and each time point in the TLV and non-TLV groups. The abbreviations are the same as in Figure 1. * *p* < 0.05 for TLV group vs. non-TLV group.

**Figure 3:**
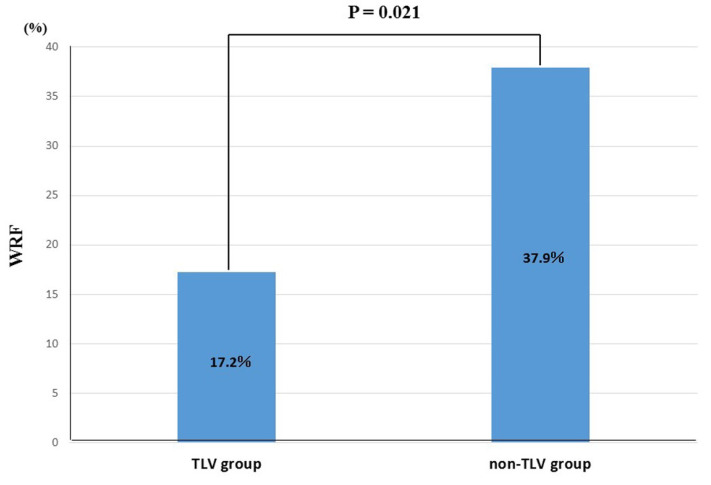
Incidence of worsening renal failure between the TLV and non-TLV group. WRF, worsening renal failure.

In the matched cohort, we investigated the renoprotection effect using the change in the Cr and eGFR levels in this study as previously mentioned. The change in the Cr level was significantly lower in the TLV group than in the non-TLV group at five days after admission but the differences in the change grade between the two groups decreased gradually over one year (Figure 2A). Regarding the eGFR, the change in the eGFR level was significantly higher in the TLV group than in non-TLV group at five days after admission but as with Cr level, the differences in the change grade between the two groups decreased gradually over one year (Figure 2B). With regard to a WRF, the incidence of a WRF was significantly lower in the TLV group than non-TLV group (17.2% vs. 37.9%, *p* = 0.021) (Figure 3).

### 3.4. Clinical Outcome

**Figure 4:**
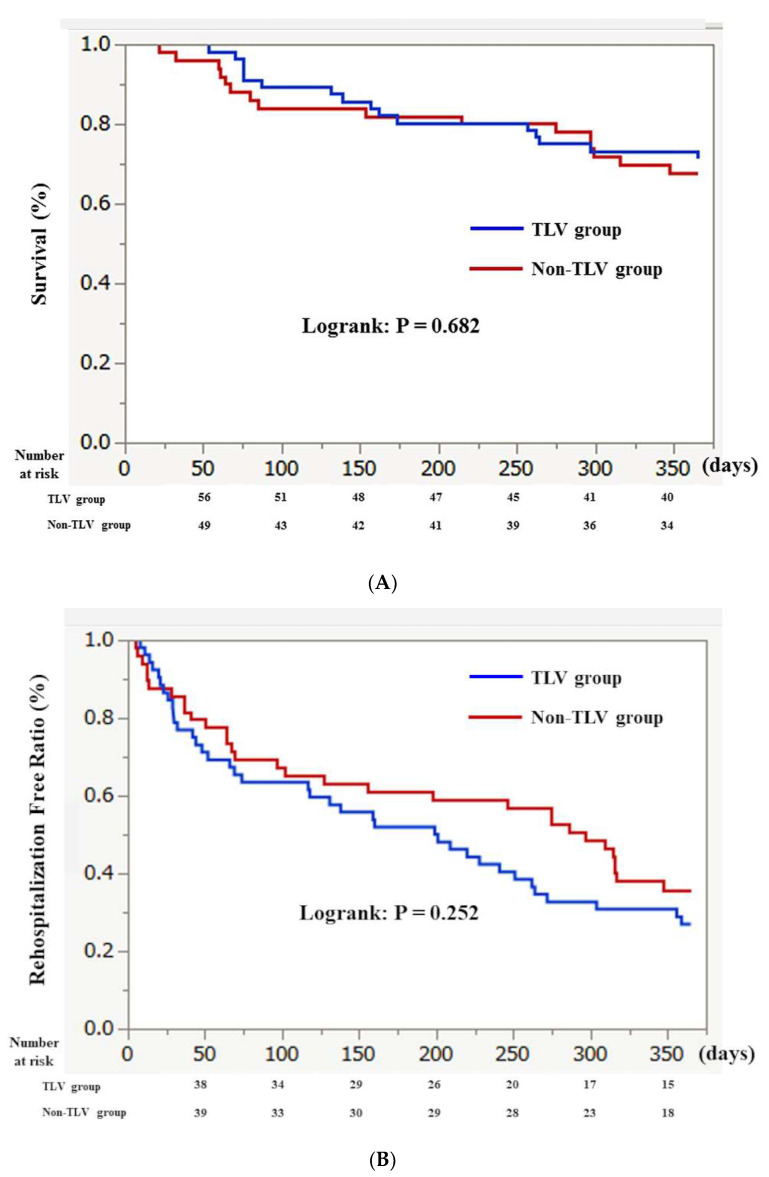
(**A**): Kaplan–Meier curve for the survival ratio between the TLV and non-TLV group in a propensity score-matched population. (**B**): Kaplan–Meier curve for the rehospitalization free ratio between the TLV and non-TLV group in a propensity score-matched population. The abbreviations are the same as in Figure 1.

In the matched cohort, a Kaplan–Meier analysis showed that the survival in the TLV group and the non-TLV group was similar over one year (72.4% vs. 70.7%, *p* = 0.682) (Figure 4A). In addition, the rehospitalization-free curve did not significantly differ between the TLV group and non-TLV group over one year (32.7% vs. 48.2%, *p* = 0.252) (Figure 4B).

## 4. Discussion

The present study has highlighted that (1) the renoprotective effect of TLV was significant in the acute phase but was weak in the chronic phase (one year), which was a temporal change, and (2) TLV did not contribute to alleviating the all-cause mortality and HF rehospitalizations over one-year. Accordingly, TLV was effective for short-term use, especially when expecting a renoprotective effect, but was not useful for long-term use considering the one-year all-cause mortality, HF rehospitalizations, and discontinuation of the renoprotective effect of TLV.

### 4.1. Temporal Change in Renoprotective Effect of TLV

In the present study, a propensity matching analysis was performed to adjust for the patient background as much as possible. After the propensity matching analysis, all the patients in the matched cohort received loop diuretics. Therefore, the results of this study can reflect the effect of adding TLV on loop diuretics.

Several previous studies have shown that poor clinical outcomes after hospitalization were found in ADHF patients with WRF [13,14]. It has been reported that although transient, WRF is associated with a longer hospitalization and higher risk of death and readmissions irrespectively of the baseline renal function [15]. Thus, it may be important to reduce WRF even in the acute phase to manage ADHF.

It has been reported that TLV is associated with a significantly lower index of WRF than conventional loop-diuretic therapy partially due to the renoprotection effect of TLV in the acute phase [4,16,17]. We also revealed that TLV could reduce a WRF in the acute phase. In addition, our results have shown a temporal change in the renoprotective effect of TLV. TLV has a significant renoprotective effect during the acute phase of ADHF, but the grade of the protection may decrease over one year.

The causative mechanism of a temporal change in the renoprotective effect of TLV is partially explained as follows. It has been reported that adding TLV to loop diuretics preserves the renal function as compared to only loop diuretics in the acute phase (within 48 h after admission), suggesting that the renal perfusion is maintained in patients with TLV [15,16,18]. Loop diuretics activate the tubuloglomerular feedback mechanism, which causes vasoconstriction of the afferent arterioles and a reduction in the renal blood flow [19]. Thus, loop diuretics occasionally raise an excessive fluid loss by a reduction in the intravascular volume, which may be correlated with a higher incidence of rehospitalizations and mortality [20,21]. On the other hand, TLV reduces the interstitial fluid more than the intravascular volume, which alleviates congestion without a reduction in the renal blood flow or activation of the renin-angiotensin system and sympathetic nervous system [18]. Thereby, in the matched cohort, the TLV group (adding TLV on to loop diuretics) can protect from a deterioration of the renal function in the acute phase (within five days after the admission) of ADHF patients as compared to the non-TLV group (only loop diuretics), but in the chronic phase, the balance of the interstitial and intravascular fluid alleviates and both volumes gradually decrease even in the TLV group, which might weaken the renoprotective effect and induce a temporal change in the renoprotective effect of TLV.

### 4.2. Effect of TLV on One-Year All-Cause Mortality and Rehospitalizations

Our results have revealed that after a propensity matching analysis, TLV did not improve the one-year all-cause mortality and rehospitalizaions. Actually, the first randomized trial of TLV, EVEREST, showed no effect of TLV on the long-term mortality or heart failure-related morbidity in ADHF patients [6]. In addition, recently, the TACTIS-HF trial [22] and SECRET trial [23] also revealed no beneficial effects of TLV on managing HF as compared to placebos. However, in Japan, many HF patients receive long-term TLV therapy [3], probably considering the favorable clinical response of TLV experienced by clinicians and approval when an adequate response is not obtained by other diuretics since December in Japan [24]. Recent long-term studies using TLV in Japan revealed relatively favorable TLV effects on HF patients, but in very limited patients who had many high-risk factors for rehospitalizations or who were possible TLV responders whose urine osmolality was ≥350 mOsm/L, which was a predictive marker of TLV responders [4,5,7]. Thus, in general, the long-term effect of TLV in HF patients cannot be sufficiently expected. Our findings of a temporal change in the renoprotective effect of TLV may be correlated with no beneficial long-term effect of TLV in HF patients.

### 4.3. Optimal Duration and Suitable Dose of TLV

The SMILE study, a post-marketing surveillance of tolvaptan, demonstrated from data of consecutive patients who had received TLV between 2011 and 2015 that 43.6% of the patients continued TLV therapy for over two weeks in the real-world clinical practice [25]. However, in other words, approximately half of the HF patients discontinued receiving TLV within two weeks, even in Japan. According to our data, the renoprotective effect of TLV on ADHF patients continued for approximately 14 days (before the discharge). In addition, our data and many other studies [6,22,23] have not shown a favorable effect of TLV on one-year mortality and rehospitalizations. Thus, the spot use of TLV (e.g., two weeks-use or during hospitalization) may be one of the favorite strategies when we use TLV for ADHF patients.

Regarding the optimal dose of TLV, a low dose of TLV may be favorable. As compared to the trials abroad using TLV, including the TACTICS-HF trial [22] and SECRET trial [23], the dose of the TLV in the Japanese studies [5,26] was lower. The dose of TLV in the former was 30 mg/day, but that in the latter was 7.5–15 mg/day. Considering the renal function [26], 7.5 mg–15 mg/day of TLV may be recommended as an optimal dose. Accordingly, the spot use of TLV (7.5 mg–15 mg/day) during hospitalization may be one of the best methods for taking the temporal change in the renoprotective effect of TLV into consideration.

### 4.4. Clinical Implications

Several studies, including our data, have shown that although TLV had favorable effects, including a renoprotective effect in the acute phase of ADHF, TLV could not show any favorable long-term clinical outcomes [6,22,23]. Although several Japanese reports showed a favorable long-term effect on very limited HF patients [7,8,27], it may not be valuable in the clinical settings because these data cannot apply to all ADHF patients. In addition, recently, the guideline-based medical therapy (GDMT) for HF included sodium–glucose cotransporter (SGLT) 2 inhibitors [28] because SGLT2 inhibitors have been to have a strong clinical impact on HF [29,30]. Thus, currently, ADHF patients usually receive SGLT2 inhibitors. SGLT2 inhibitor’s ability is to selectively reduce the interstitial fluid [31], which is similar to TLV. These effects of SGLT2 inhibitors and TLV are unique as compared to the traditional diuretics, including loop diuretics that cause a reduction in the intravascular volume. In addition, SGLT2 inhibitors have multiple beneficial cardioprotective effects other than the diuretic effects, which may affect the favorable long-term clinical outcomes [31]. TLV and SGLT2 inhibitors have different mechanisms. SGLT2 inhibitors act on the proximal tubular site and decrease the interstitial volume without a neurohormonal response [32]. On the other hand, TLV blocks the arginine vasopressin (AVP)-aquaporin-2 pathway between the apical plasma membrane and subapical vesicles in the principal cells of the collecting duct and increases the excretion of electrolyte-free water in urine [33].

When ADHF patients were admitted, they usually received SGLT2 inhibitors and loop diuretics as a GDMT. Hence, TLV can be effective as another useful drug to reduce the interstitial fluid because SGLT2 inhibitors and TLV have different mechanisms and act on different sites even though these medications have similar effects that selectively reduce the interstitial fluid. Therefore, TLV may be useful for a spot-use (e.g., two weeks use or during hospitalization) for ADHF and not be adequate for the long-time use because our data have shown a temporal change in the renoprotective effect of TLV, and there have been little data on TLV showing the long-term beneficial clinical outcomes.

### 4.5. Study Limitations

First, the present study was a single-center, non-randomized registry-based study. However, our study revealed a temporal change in TLV with the long time use, which is valuable information. To confirm our findings, further prospective large-scale studies are required. Second, we tried to perform adjustments of the patient background between the TLV and non-TLV groups, but several factors could not be adjusted. In addition, there may have been residual and unmeasured confounders that could not be ruled out. This might have affected our results. Finally, in this study, the dose of TLV was determined by each physician, considering the stability of the patient hemodynamics and the degree of congestion that may have involved a bias in selecting the dose of TLV.

## 5. Conclusions

TLV, an oral selective vasopressin 2 receptor antagonist, is a unique drug that selectively reduces the interstitial fluid, while loop diuretics reduce the intravascular volume. Our data showed a temporal change in the renoprotective effects of TLV, which decreased after two weeks post-admission, and no beneficial long-term effect of TLV for HF. Thus, one of the suitable methods for using TLV for ADHF may be a spot use of TLV (two weeks use or during hospitalization). In the recent GDMT era, which recommends SGLT2 inhibitors, the spot use of TLV can enable the management of ADHF because both TLV and SGLT2 inhibitors have relatively similar diuretic effects, which reduce the interstitial fluid, but different mechanisms and acting sites.

## Figures and Tables

**Table 1:** Patient Characteristics of Overall and Propensity Score-Matched Cohort.

	Overall			Propensity Score-Matching		
	TLV Group (n = 129)	Non-TLV Group (n = 756)	*p* Value	TLV Group (n = 58)	Non-TLV Group (n = 58)	*p* Value
**Clinical data**						
Age, years	79 (73–86)	79 (71–85)	0.594	79 (74–86)	77 (70–85)	0.338
Male, n (%)	85 (62.0)	398 (70.0)	0.026	34 (58.6)	29 (50)	0.456
Hypertension, n (%)	79 (57.7)	542 (63.2)	0.005	26 (44.8)	43 (74.1)	0.002
Diabetes mellitus, n (%)	56 (40.9)	286 (37.0)	0.390	25 (43.1)	23 (39.7)	0.851
Dyslipidemia, n (%)	42 (30.7)	273 (356.3)	0.286	21 (36.2)	26 (44.8)	0.450
Chronic kidney disease, n (%)	105 (76.7)	369 (47.7)	<0.001	42 (72.4)	34 (58.6)	0.171
Smoker, n (%)	65 (47.0)	339 (43.8)	0.456	22 (37.9)	22 (37.9)	1.000
Past history of HF admission, n (%)	109 (73.6)	328 (42.4)	<0.001	48 (82.8)	35 (60.3)	0.013
Length of stay, days	20 (13–26)	16 (12–24)	<0.001	18 (13–23)	17 (14–25)	0.862
Systolic blood pressure, mmHg	117 (100–133)	126 (108–149)	0.028	115 (99–130)	115 (102–130)	0.330
Diastolic blood pressure, mmHg	62 (51–69)	63 (56–72)	0.018	65 (55–79)	71 (64–82)	0.040
Body weight, kg	53 (33–57)	56 (47–66)	0.170	55 (47–63)	54 (48–64)	0.993
**Electrocardiographic data at discharge**						
Heart rate	73 (65–88)	80 (71–95)	<0.001	73 (62–88)	73 (66–83)	0.067
AF, n (%)	48 (35.0)	231 (29.8)	0.359	19 (32.8)	17.1 (29.3)	0.841
**Laboratory data at discharge**						
CRP, mg/L	0.36 (0.16–1.02)	0.47 (0.18–1.46)	0.094	0.40 (0.12–0.88)	0.55 (0.20–1.95)	0.639
BNP, pg/mL	1298 (628–1570)	809 (426–1329)	<0.001	947 (532–1511)	1004 (548–1695)	0.178
Hemoglobin, g/dL	8.1 (7.0–9.6)	11.1 (9.8–13.0)	0.003	10.7 (9.4–12.5)	10.8 (9.28–12.10)	0.865
Creatinine, mg/dL	1.54 (1.23–2.19)	1.10 (0.86–1.81)	<0.001	1.51 (1.29–2.01)	1.47 (0.996–2.28)	0.489
eGFR, mL/min/1.73 m^2^	28.3 (20.2–39.8)	33.6 (22.1–56.2)	0.004	28.5 (22.7–39.9)	30.7 (21.56–47.7)	0.628
Albumin, g/dL	3.7 (3.4–3.9)	3.5 (3.2–3.8)	0.121	3.7 (3.4–3.9)	3.5 (3.3–3.9)	0.121
Sodium, mEq/L	138 (134–140)	140 (137–142)	<0.001	137 (134–140)	139 (137–141)	0.058
Potassium, mEq/L	4.5 (3.7–4.6)	4.2 (3.8–4.7)	<0.001	4.5 (4.1–4.8)	4.2 (3.8–4.8)	0.355
**Echocardiographic data at discharge**						
LVDd, mm	55 (47–64)	52 (47–58)	0.030	58 (46–55)	53 (43–62)	0.661
LVDs, mm	43 (30–54)	38 (30–49)	0.030	46 (29–55)	39 (31–53)	1.000
LVEF, %	42 (31–63)	51 (37–65)	0.012	44 (33–65)	51 (31–63)	0.792
LAD, mm	51 (46–55)	49 (45–53)	0.121	52 (47–55)	51 (44–56)	0.618
E/e’	17.4 (13.8–23.3)	17.8 (13.3–23.3)	0.620	17.7 (14.3–27.3)	17.5 (13.7–23.1)	0.635
MR, n (%)	11 (8.0)	115 (14.9)	0.031	8 (13.8)	9 (15.5)	1.000
AR, n (%)	11 (8.0)	38 (4.9)	0.149	6 (10.3)	3 (5.2)	0.490
TR, n (%)	22 (16.1)	84 (10.9)	0.084	10 (17.2)	13 (22.49)	0.642
**Medication at discharge**						
β blocker, n (%)	103 (75.2)	492 (63.4)	0.008	45 (77.6)	44 (75.9)	1.000
ACEI/ARB, n (%)	64 (46.7)	448 (57.9)	0.019	22 (37.9)	38 (65.5)	0.005
MRA, n (%)	73 (53.3)	309 (39.9)	0.005	33 (56.9)	29 (50.0)	0.577
Loop diuretics, n (%)	133 (97.0)	597 (77.1)	<0.001	58 (100.0)	58 (100.0)	1.000
Loop diuretics dose (mg/day)	60 (40–80)	30 (20–60)	<0.001	60 (40–80)	40 (23–80)	0.093
